# Rho and Rab Family Small GTPases in the Regulation of Membrane Polarity in Epithelial Cells

**DOI:** 10.3389/fcell.2022.948013

**Published:** 2022-07-04

**Authors:** Klaus Ebnet, Volker Gerke

**Affiliations:** ^1^ Institute-Associated Research Group: Cell Adhesion and Cell Polarity, Institute of Medical Biochemistry, ZMBE, University of Münster, Münster, Germany; ^2^ Interdisciplinary Clinical Research Center (IZKF), University of Münster, Münster, Germany; ^3^ Cells-In-Motion Cluster of Excellence (EXC1003-CiM), University of Münster, Münster, Germany

**Keywords:** apico-basal membrane polarity, Rab small GTPase, Rho small GTPase, tight junctions, vesicle transport

## Abstract

Membrane polarity, defined as the asymmetric distribution of lipids and proteins in the plasma membrane, is a critical prerequisite for the development of multicellular tissues, such as epithelia and endothelia. Membrane polarity is regulated by polarized trafficking of membrane components to specific membrane domains and requires the presence of intramembrane diffusion barriers that prevent the intermixing of asymmetrically distributed membrane components. This intramembrane diffusion barrier is localized at the tight junctions (TJs) in these cells. Both the formation of cell-cell junctions and the polarized traffic of membrane proteins and lipids are regulated by Rho and Rab family small GTPases. In this review article, we will summarize the recent developments in the regulation of apico-basal membrane polarity by polarized membrane traffic and the formation of the intramembrane diffusion barrier in epithelial cells with a particular focus on the role of Rho and Rab family small GTPases.

## 1 Introduction

Epithelia and endothelia form sheets of cells which separate different tissue compartments and which segregate the organism’s interior from the external environment. Individual cells embedded in these sheets are connected to each other by cell-cell junctions. Cell-cell junctions not only integrate individual cells in the cellular sheet but also separate the plasma membrane of each cell into separate domains, a membrane domain that faces the free space (typically the lumen of an organ) and that is defined as the apical membrane domain, and a bounded membrane domain that is in contact with either another cell or the extracellular matrix and that is defined as the basolateral membrane domain. Apical membrane domains regulate the absorption of materials and, in case of endothelial cells, the transient interaction with cells of the immune system, whereas basolateral membrane domains regulate the integrity of the cellular sheet, the response to mechanical forces during morphogenetic processes or during collective cell migration, and the resistance of the sheets towards physical impact. Consequently, apical and basolateral membrane domains differ in their composition of integral membrane proteins and lipids, a phenomenon which is commonly referred to as apico-basal membrane polarity.

Apico-basal polarization requires the presence of an intramembrane diffusion barrier which prevents the intermixing of freely diffusible membrane components between the two membrane compartments. This is particularly important for lipids, which in contrast to integral membrane proteins are mostly not embedded in larger complexes or clusters connected to the actin cytoskeleton, and are thus more mobile. In vertebrates, the diffusion barrier is localized at the tight junctions (TJ), a structure at the most apical region of cell-cell junctions (Tsukita et al., 2001). TJs are characterized by close appositions of the membranes of two adjacent cells, which on freeze-fracture electron micrographs appear as anastomosing intramembrane particle strands (Farquhar and Palade, 1963; Claude and Goodenough, 1973). The particle strands are generated by proteins of the claudin family, which multimerize in cis and trans to form a mesh-like structure (Gunzel and Yu, 2013).

TJs contain a large number of proteins including integral membrane proteins like claudins, Marvel proteins and junctional adhesion molecules (JAMs), peripheral membrane proteins like zonula occludens (ZO) proteins, partitioning-defective (PAR) proteins, Protein associated with Lin-7 1 (Pals1) and Pals-1-associated tight junction protein (PATJ), but also adapter proteins, heterotrimeric G-proteins and small GTPases and their regulators, and kinases and phosphatases (Zihni et al., 2016; Tan et al., 2020). Many of these proteins are assembled in specific proteins complexes, like the Crumbs (CRB) complex or the partitioning-defective (PAR)—aPKC complex. The abundance of PDZ domain-containing scaffolding proteins indicates that TJs are sites of intensive signalling activities, and that their function in regulating the permeability of cellular sheets is subject to dynamic and sophisticated regulation.

Early studies suggested that the TJs act both as a barrier to the diffusion of small solutes across the paracellular pathway (paracellular gate function) (Goodenough and Revel, 1970) and as a barrier to the diffusion of intramembrane proteins and lipids (molecular fence function) (Dragsten et al., 1981). These two functions seem to be regulated by different molecular mechanisms. While in the absence of claudins or in the absence of the claudin-scaffolding zonula occludens (ZO) proteins the barrier function is lost, the fence function is retained under these conditions (Umeda et al., 2006; Otani et al., 2019). Thus, the gate and the fence functions of TJs reside in the same subcellular structure but differ in their molecular nature.

After the establishment of a diffusion barrier at the TJs, targeted vesicle transport to the apical and basolateral membrane domains is required to generate and maintain membrane identity. This is achieved by selective anterograde transport to the two principal membrane domains and by unique recycling pathways (Nelson and Yeaman, 2001; Ang and Folsch, 2012).

**FIGURE 1:**
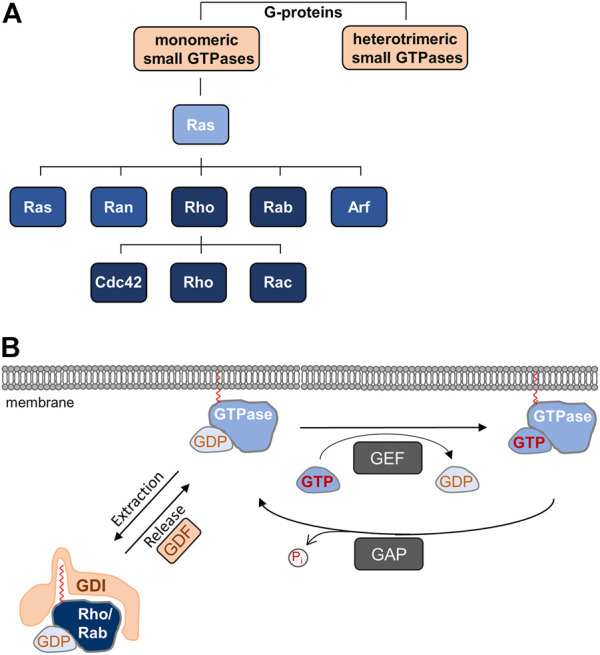
**(A)** Monomeric small GTPases belong to G-proteins. Ras is the founding member of the Ras superfamily of monomeric small GTPases, which is divided in five families. The Rho family is further subdivided into the Cdc42, Rho, and Rac subfamilies. **(B)** Cyclic regulation of monomeric small GTPases. Monomeric GTPases are anchored in membranes through prenyl groups. Local guanine nucleotide exchange factors (GEFs) catalyze the exchange of GDP by GTP resulting in the active GTPase, whereas local GTPase-activating proteins (GAPs) hydrolyze GTP to release inorganic phosphate (P_i_), which results in the inactivation of the GTPase. Rho and Rab family GTPases are sequestered in the GDP-bound, inactive form to the cytosol by guanine nucleotide dissociation inhibitors (GDIs), which mask the prenyl groups required for membrane insertion. Through the activities of GDI displacement factors (GDFs), inactive GTPases are released from GDI-inhibition allowing membrane localization.

TJs are subject to dynamic regulation in physiological and pathological situations. Dynamic cellular processes are frequently regulated by monomeric small GTPases, a superfamily of proteins which bind and hydrolyze GTP, and which switch between inactive and active states by binding GDP or GTP, respectively (Bourne et al., 1990) (Figure 1). Based on sequence homology and functional similarity the GTPase superfamily, which contains more than 150 members, is subdivided in five families, the Ras, Rho, Rab, Ran, and Arf families (Kahn et al., 1992) (Figure 1A). While the functions of these families do overlap to some extent, the Rho family GTPases regulate cell morphology through their activities on the actin cytoskeleton, whereas the Rab and Arf families are important regulators of vesicle trafficking (Jaffe and Hall, 2005; Goitre et al., 2014). In this review article, we describe the role of Rho and Rab family small GTPases in the regulation of apico-basal membrane polarity through their functions during cell-cell contact formation and in directed vesicle transport. We will focus on the role of these small GTPases during key processes regulating apico-basal membrane polarity in vertebrate epithelial cells. For the role of Ras and Arf family monomeric small GTPases in polarity, we refer the reader to recent reviews (Young and Rodriguez-Viciana, 2018; Mima, 2021).

## 2 Rho and Rab Family Small GTPases

All GTPases have in common that their activity is regulated by guanine nucleotide exchange factors (GEFs) and GTPase-activating proteins (GAPs) (Mosaddeghzadeh and Ahmadian, 2021) (Figure 1). GEFs catalyze the dissociation of GDP thus allowing the binding of GTP, which results in the active form of the GTPase and binding to its effector proteins. GAPs stimulate the intrinsic activity of the proteins to hydrolyze GTP to GDP, leading to the inactive form of the GTPase (Cherfils and Zeghouf, 2013). A second commonality of GTPases is the posttranslational addition of lipid moieties consisting of either three (farnesyl) or four (geranylgeranyl) isoprene units, a process referred to as prenylation. In Rho GTPases, the prenyl groups are attached to the cysteine residue present in the CAAX motif, whereas in Rab GTPases, the prenyl groups are attached to C-terminal Cys residues (Muller and Goody, 2018; Brandt et al., 2021). The prenyl groups anchor the GTPases in lipid bilayers, for example in the plasma membrane or in endomembranes, where they are activated by locally resident GEFs (Hodge and Ridley, 2016). Most GTPases depend on prenylation and membrane localization for function. Membrane targeting of Rho and Rab GTPases is antagonized by a third family of GTPase regulators, guanine nucleotide dissociation inhibitors (GDIs). GDIs binding to inactive (GDP-bound) Rho and Rab GTPases masks the prenyl group, thus blocking membrane insertion and promoting their sequestration to the cytosol. GDI binding also protects GTPases from degradation (Garcia-Mata et al., 2011; Muller and Goody, 2018) (Figure 1). At any given time, only a small fraction of all Rho GTPases is associated with membranes. The vast majority is maintained in the cytosol through GDIs (Garcia-Mata et al., 2011). As opposed to GEFs and GAPs, GDIs exist in a limited number with only three members (RhoGDI-1, -2, -3, RabGDIα, -β, -3) identified so far (Nazlamova et al., 2017; Muller and Goody, 2018; Ahmad Mokhtar et al., 2021).

## 3 Rho Family Small GTPases in Membrane Polarity

### 3.1 Rho Small GTPases During Early Cell-Cell Contact Formation

Given the critical role of TJs in membrane polarity, it is important to understand the process of cell-cell contact and TJ formation. When migrating epithelial cells encounter other cells through cellular protrusions, they first engage in initial cell-cell contacts called “puncta” or “primordial, spot-like adherens junctions” (pAJs) (Yonemura et al., 1995). These puncta localize at the tips of F-actin-rich protrusions and are positive for several cell-cell adhesion receptors including E-cadherin, Nectin-2, and Junctional Adhesion Molecule (JAM)-A, as well as for cytoplasmic scaffolding proteins associated with cell adhesion receptors, including α-catenin, β-catenin, ZO-1 and Afadin (Yonemura et al., 1995; Ando-Akatsuka et al., 1999; Asakura et al., 1999; Ebnet et al., 2001; Suzuki et al., 2002). Molecules that are localized separated from each other at TJs and AJs in fully polarized epithelial cells, co-localize at pAJs at this early stage of junction formation. The next step in the maturation process involves the activation of Rho GTPases as a direct consequence of cell-cell adhesion. Several adhesion receptors that are localized at pAJs can activate Rho family small GTPases, including E-cadherin (Ehrlich et al., 2002; Yamada and Nelson, 2007), Nectins (Kawakatsu et al., 2002), and JAM-A (Tuncay et al., 2015), and the importance of Rho family GTPases in the regulation of cell-cell contact formation is widely documented (Arnold et al., 2017; Cerutti and Ridley, 2017; Braga, 2018). A critical step in the generation of membrane polarity, however, is the maturation of immature cell-cell junctions to mature cell-cell junctions with TJs being separated from AJs. This step requires the activation of atypical protein kinase C (aPKC) mediated by Rac1 and/or Cdc42.

Atypical PKC is part of a highly conserved polarity protein complex, the partitioning-defective (PAR)—aPKC complex (Suzuki and Ohno, 2006). The PAR—aPKC complex regulates various aspects of cell polarity including apico-basal membrane polarity in epithelial cells, anterior-posterior polarity in the *C.elegans* zygote, or the specification of the axon in neurons (Suzuki and Ohno, 2006; Iden and Collard, 2008). In epithelial cells, aPKC exists in a ternary complex with the polarity proteins PAR-3 and PAR-6, which both directly interact with aPKC (Ohno, 2001). In this complex, aPKC is maintained in an inactive conformation. The binding of active Cdc42 or active Rac1 to PAR-6 induces a conformational change of PAR-6 that releases aPKC from PAR-6 inhibition (Yamanaka et al., 2001). Active aPKC then phosphorylates a number of substrates including PAR-3 and PAR1 (Nagai-Tamai et al., 2002; Suzuki et al., 2004), which results in their separate localization at TJs and at the basolateral membrane domain, respectively. Of note, in the absence of aPKC kinase activity, cells are able to form pAJs but fail to develop belt-like AJs and TJs (Suzuki et al., 2001; Suzuki et al., 2002). The activation of aPKC by Rho GTPases Cdc42 and/or Rac1 is thus a key step in the development of membrane asymmetry in polarized epithelial cells.

Many studies that address the role of RhoGTPase regulation in TJ formation and maintenance focus on actomyosin-driven contractility and the paracellular permeabilty of TJs. However, since the two principal functions of TJs, i.e., gate and fence function are regulated through distinct molecular mechanisms (Umeda et al., 2006), it is well possible that Rho family regulators involved in the regulation of TJ formation or maintenance may selectively affect one of the two principal functions of TJs.

### 3.2 Rho Small GTPases in the Maintenance of Membrane Asymmetry

**FIGURE 2:**
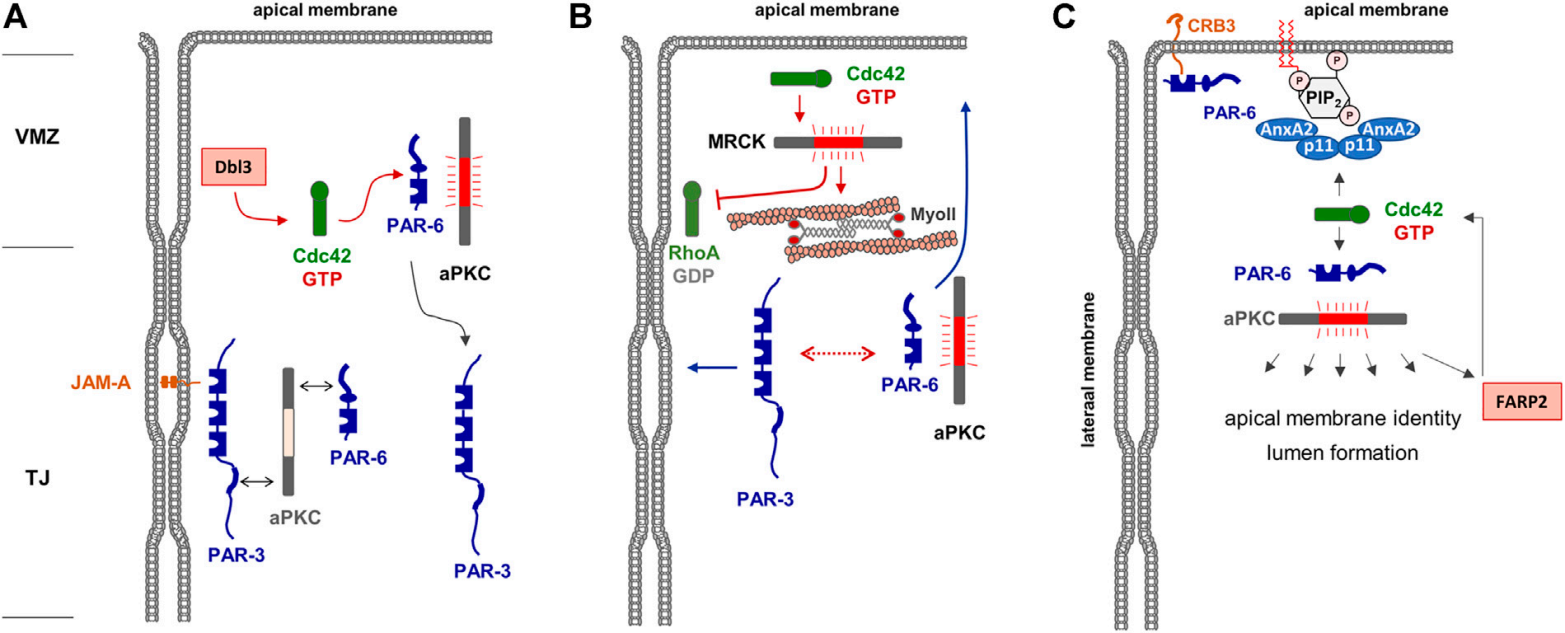
Regulation of apical membrane identity in polarized epithelial cells. **(A)** The Par—aPKC complex is localized at the TJs, most likely through PAR-3 interacting with JAM-A. The Cdc42 GEF Dbl is localized at the vertebrate marginal zone (VMZ) and activates Cdc42, which in turn interacts with PAR-6 and induces a conformational change of PAR-6 that allows activation of aPKC. Phosphorylation of PAR-3 results in the dissociation of PAR-3. Note that this is a dynamic cycle, and that the stable association of the heterotrimeric PAR-3—aPKC - PAR-6 complex may be short-lived. **(B)** Active Cdc42 activates MRCK which stimulates actomyosin contractility-mediated segregation of PAR-3 and PAR-6—aPKC (dotted line with double arrows) to lateral and apical membrane domains, respectively (blue arrows). In addition, MRCK inhibits RhoA at the lateral membrane domain. **(C)** A tetrameric complex of Annexin A2 (AnxA2) and p11 is localized in the apical membrane by interacting with phosphatidyl-inositol-4,5-bisphosphate (PIP_2_). This complex recruits and activates the PAR-6—aPKC complex which phosphporylates various substrates involved in apical membrane identity and lumen formation. Among its substrates is the Cdc42 GEF FARP2, which catalyzes GDP-exchange of Cdc42, providing a possible positive feedback loop of Cdc42 activation in the apical membrane. Note that CRB3 present in the apical membrane may provide an additional anchor for PAR-6 and possibly the PAR-6—aPKC complex.

After the formation of TJs which separate apical and basolateral membrane domains, the activity of Rho GTPases is continuously required for the maintenance of membrane identity. After the activation of aPKC and the subsequent phosphorylation of PAR-3, the PAR-6—aPKC complex remains as a unit whereas PAR-3 separates from PAR-6—aPKC (Nagai-Tamai et al., 2002). In fully polarized epithelial cells, PAR-6—aPKC segregates into the apical domain whereas PAR-3 localizes to the TJs. Apical membrane localization is particularly evident when cells are grown under three-dimensional culture conditions embedded in extracellular matrix (Durgan et al., 2011). Under these conditions, polarized epithelial cells form cysts, hollow spheres consisting of a single layer of epithelial cells which surround a single lumen (O'Brien et al., 2002). In cells grown to cysts, PAR-6 and aPKC are highly enriched in the lumen-facing apical membrane domain whereas PAR-3 is excluded from the apical membrane (Durgan et al., 2011). Although the formation of a ternary PAR-3—aPKC—PAR-6 complex is required for the development of apico-basal membrane polarity (Horikoshi et al., 2009), it has long been unclear by which mechanisms the separation of PAR-6—aPKC from PAR-3 is regulated, and how this separation into different membrane domains is maintained. Studies in Drosophila follicle epithelial cells already indicated that PAR-6—aPKC localize above PAR-3/Bazooka in the so-called marginal zone, and that the phosphorylation of PAR-3 by aPKC excludes PAR-3 from the apical domains (Morais-de-Sa et al., 2010). More recent studies in vertebrate epithelial cells showed that a similar mechanism operates in vertebrate epithelial cells and that Cdc42 is a central component of this mechanism. Cdc42 is activated at the border between the cell-cell contacts and the contact-free apical membrane domain, the vertebrate marginal zone (VMZ), through the activity of the Cdc42 GEF Dbl3 (Zihni et al., 2014). Locally active Cdc42 can bind to PAR-6 triggering the activation of PAR-6-/PAR-3-associated aPKC resulting in PAR-3 phosphorylation and its segregation to the lateral membrane domain (Zihni et al., 2014) (Figure 2A). Through an additional pathway that involves the Cdc42-mediated activation of the Rho kinase (ROCK)-related myotonic dystrophy kinase-related Cdc42-binding kinase (MRCK), apical Cdc42 stimulates apical myosin II activation and junctional RhoA inhibition, thereby mediating actomyosin contractility-mediated PAR protein segregation (Zihni et al., 2017), a mechanism that has also been described in the regulation of PAR protein asymmetry in the *C. elegans* zygote (Munro et al., 2004) (Figure 2B). MRCK-regulated actomyosin contractility appears to emerge as a more general regulator of membrane specification (Zihni, 2021). By activating aPKC at the marginal zone, Cdc42 thus triggers a biochemical and a mechanical mechanism of PAR protein segregation to regulate the positioning of the apical-lateral border and the specification of the apical and basolateral membranes, which defines Cdc42 as a central regulator of apico-basal membrane polarity in epithelial cells. Interestingly, recent findings in the Drosophila follicular epithelium identified the Cdc42 GAP RhoGAP19D at the lateral membrane domain of follicular epithelial cells (Fic et al., 2021). RhoGAP190D mutants lead to Cdc42 activity at the lateral membrane, which results in lateral contractility through the activity of the MRCK orthologue Genghis khan (Gek), and expansion of the apical domain through increased PAR-6—aPKC activity (Fic et al., 2021). These observations provide a mechanism to inhibit the activity of Cdc42 at the lateral membrane domain and further underline the role of MRCK in apical membrane specification.

Studies with 3D-cultured MDCK cells further supported that Cdc42 activity is continuously needed at the apical membrane domain. Cdc42 interacts with annexin A2 (AnxA2) localized in the apical membrane in a GTP-dependent manner (Martin-Belmonte et al., 2007). Apical membrane specification is regulated by the lipid phosphatase PTEN present in the apical membrane, which mediates enrichment of PtdIns(4,5)P_2_ at this membrane compartment. AnxA2 binding to PtdIns(4,5)P_2_ mediates the specific enrichment of active Cdc42 in the apical membrane domain. Active Cdc42 then binds and recruits the PAR-6—aPKC module to the apical membrane, which is necessary for lumen formation. More recent findings indicate that aPKC interacts with and phosphorylates the Cdc42 GEF FARP2, and that FARP2 activity is required for apico-basal polarity and a functional barrier in polarized Caco2 cells (Elbediwy et al., 2019) suggesting that Cdc42 may also act downstream of aPKC, which would represent a positive feedback loop in the regulation of apical membrane identity and TJ formation (Figure 2C).

The activity of Rac1 is regulated in a different way. During the process of MDCK cyst formation, Rac1 activity is downregulated at the apical membrane domain relative to the basolateral membrane domain (Yagi et al., 2012b). Ectopic activation of Rac1 at the apical membrane in mature cysts disturbs TJs and mislocalizes polarity proteins such as syntaxin-4, which localizes to the basolateral domain in unperturbed cells (Yagi et al., 2012b). Inactivation of Rac1 is most likely mediated by the Rac1-specific GAPs chimaerin (CHN)-1 (ARHGAP2) and CHN-2 (ARHGAP3), which localize to the apical membrane domain through their interaction with diacylglycerol (Yagi et al., 2012a). The enrichment of chimaerins CHN-1 and CHN-2 at the apical membrane thus suppresses Rac1 activity at the apical membrane to maintain apico-basal membrane polarity.

### 3.3 The Apical Junctional Complex in Vertebrate Epithelial Cells and the Localization of Rho GTPases and Their Regulators

**FIGURE 3:**
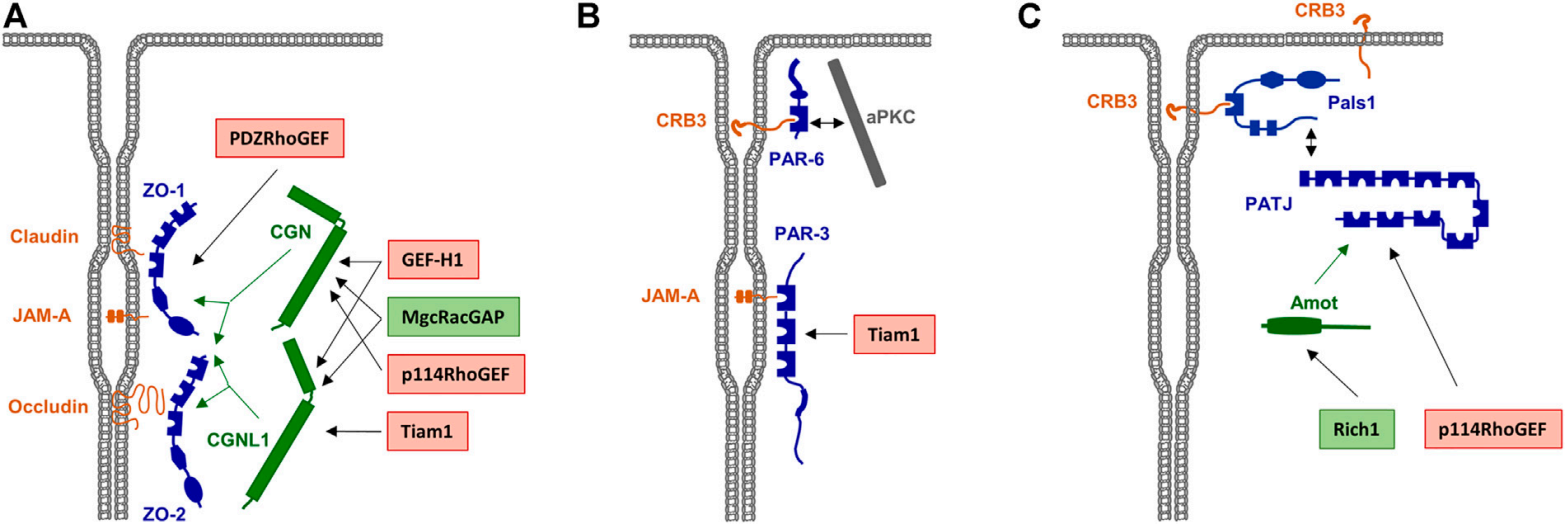
Scaffolding proteins for RhoA family GEFs and GAPs a the TJs. **(A)** Rho family GEFs and GAPs associated with the Zonula occludens (ZO) complex. ZO-1 and ZO-2 directly interact with various integral membrane proteins like Occludin, Claudins and JAM-A. Rho family GEFs and GAPs can directly interact with the ZO complex but also indirectly through the adapter proteins cingulin (CGN) and cingulin-like 1 (CGNL1), which interact with ZO proteins as indicated by green arrows. GEFs and GAPs are indicated in red and green boxes, respectively. **(B)** RhoA GEFs associated with the PAR—aPKC complex. PAR-3 directly interacts with JAM-A. The RhoA GEF Tiam1 directly interacts with PAR-3. **(C)** Rho family GEFs and GAPs associated with the CRB3—Pals1—PATJ complex. PATJ is associated with the membrane through Pals1 and CRB3. The interaction of Rich1 with PATJ is mediated by Amot, p114RhoGEF directly interacts with PATJ.

TJs contain two conserved polarity protein complexes, the PAR—aPKC complex and the Crumbs (CRB)—Pals1—PATJ complex (shortly Crumbs complex) (Wang and Margolis, 2007) (Figure 3). As outlined in the previous section, PAR-6 and aPKC segregate from PAR-3 to occupy a region that is apical to PAR-3 in polarized epithelial cells. The Crumbs polarity complex—similar to PAR-6 and aPKC—is also part of the most apical region of interepithelial cell junctions and reaches partially into the free apical membrane domain of the epithelial cells (Lemmers et al., 2004) (Figure 3). The Crumbs complex can directly interact with PAR-6 through both CRB3 and Pals1 (Hurd et al., 2003; Lemmers et al., 2004). The interaction of PAR-6—aPKC with CRB3 is promoted by the WD40 repeat domain-containing protein Morg1 and by apically localized Cdc42 (Hayase et al., 2013). The Crumbs complex thus defines a region apically to the TJs, which in analogy to a region at cell-cell contacts of invertebrates has been named vertebrate marginal zone (Tan et al., 2020). Based on the proteomes identified at the VMZ and at the TJ area of vertebrate epithelial cells, it is likely that small GTPase signalling is involved in the formation and/or maintenance of both subregions of vertebrate TJs.

Since Rho GTPases are sequestered to the cytosol immediately after their inactivation, it has been difficult to directly demonstrate their localization at specific membranous sites. However, the identification of RhoGEFs or RhoGAPs at the TJs provides strong evidence for GTPase signalling at TJs. For example, the RhoGEFs ARHGEF2/GEF-H1 (Benais-Pont et al., 2003; Aijaz et al., 2005), ARHGEF18/p114RhoGEF (Terry et al., 2011), ARHGEF11/PDZ-RhoGEF (Itoh et al., 2012), and Tiam1 (Mack et al., 2012) have been identified at the TJ area. Also, the RhoGAPs MgcRacGAP (Guillemot et al., 2014), Rich1 (Wells et al., 2006) and ARHGAP29 (Tan et al., 2020) have been identified at the TJs and at the VMZ. As additional evidence for a TJ-specific regulation of RhoGTPase activities, several TJ-localized peripheral membrane proteins serve as scaffolds for RhoGTPase regulators. These include ZO-1 (Itoh et al., 2012), ZO-2 (Raya-Sandino et al., 2017), cingulin (CGN) and cingulin-like 1 (CGNL1/paracingulin/JACOP) (Aijaz et al., 2005; Guillemot et al., 2008; Terry et al., 2011; Guillemot et al., 2014), and the polarity proteins PAR-3 (Mack et al., 2012) and PATJ (Nakajima and Tanoue, 2011). The presence of both regulators of GTPase activity as well as of scaffolds for these regulators thus makes a strong point for a highly complex and dynamic regulation of Rho GTPase signaling at the TJs (Figure 3).

### 3.4 Regulation of Rac1/Cdc42 Activities at the Tight Junctions and at the Apical Membrane Domain

As outline before, the activity of Rho family GTPases is critical for the maturation of pAJs to polarized, mature cell-cell contacts with AJs and TJs, and this activity is most likely required for the activation of aPKC as part of the PAR—aPKC complex. A potential regulator of Rac1 activity is PAR-3, which directly interacts with the Rac1 GEF Tiam1 (Nishimura et al., 2005). Studies in both cultured primary keratinocytes and in MDCK cells showed that the binding of Tiam1 to PAR-3 regulates TJ biogenesis. In the absence of Tiam1, keratinocytes are able to form pAJs but fail to develop these immature contacts into mature cell-cell junctions, which is highly reminiscent to epithelial cells lacking aPKC (Suzuki et al., 2001). These finding strongly suggest that PAR-3-bound Tiam1 activates aPKC after initial junctions have been formed in keratinocytes. In MDCK cells, the absence of PAR-3 was found to disrupt TJ assembly with a concomitant constitutive activation of Rac1 (Chen and Macara, 2005), which can be interpreted as a negative regulatory function of PAR-3 in sequenstering Tiam1 away from Rac1 at cell-cell contacts. Since the subcellular localization of active Rac1, for example by Förster resonance energy transfer (FRET) experiments, has not been analyzed in these studies it could as well be that PAR-3 deletion results in enhanced recruitment of Tiam1 to other subcellular locations, which could also result in constitutive Rac1 activation at such sites, a scenario that would be compatible with a positive regulatory function of the PAR-3—Tiam1 complex at cell-cell junctions. Interestingly, in MDCK cells it has also been observed that a Rac1 activity gradient exists along the apical—basal polarity axis that is generated by a negative regulatory role of PAR-3 on Rac1 *via* Tiam1 at the apical region of cell-cell junctions, and a positive regulatory role of β2-syntrophin *via* Tiam1 at the subapical region of cell-cell junctions (Mack et al., 2012). Thus, it is likely that PAR-3 localized at TJs sequesters Tiam1 thereby preventing high Rac1 activity levels to facilitate the generation of a Rac1 activity gradient along cell-cell junctions (Yagi et al., 2012b; Mack et al., 2012) (Figure 3).

Rich1/ARHGAP17 is a RhoGAP for Rac1 and Cdc42 with a strong selectivity for Cdc42 in epithelial cells (Richnau and Aspenstrom, 2001). Rich1 is targeted to TJs through its association with the scaffold protein Angiomotin (Amot), which interacts with the Crumbs complex component PATJ (Wells et al., 2006). Downregulation of Rich1 accelerates the loss of the barrier functions induced by Ca^2+^ removal (Wells et al., 2006), which suggests that the maintenance of functional TJs requires that the levels of active Cdc42 at TJs are kept low. Interestingly, observations in HEK293 cells and MDCK cells also indicate that Merlin, the protein encoded by the neurofibromatosis type 2 (NF2) tumor suppressor gene and a regulator of Hippo signalling (Zheng and Pan, 2019), is part of the Amot—PATJ—Pals1 complex and directly interacts with Amot in a competitive manner with Rich1 to regulate Rac1 activity (Yi et al., 2011). These fndings suggested that also the activity of Rac1 at the TJs may be subject to regulation by Rich1.

MgcRacGAP/RacGAP1 is a RhoGAP with strong activity towards Cd42 and Rac1 and weak activity towards RhoA (Toure et al., 1998). MgcRacGAP is enriched at the apical junctional complex of *Xenopus* epithelial cells (Breznau et al., 2015) and of MDCK cells (Guillemot et al., 2014). Its recruitment to TJ can be mediated by both CGN and CGNL1, which both directly interact with MgcRacGAP (Guillemot et al., 2014) (Figure 3). The localization of MgcRacGAP at the TJ further indicates that Cdc42 and Rac1 activities must be kept at low levels there to maintain the epithelial barrier function.

### 3.5 Regulation of RhoA Activity at the Tight Junctions

GEF-H1/ARHGEF2 is a GEF for RhoA which localizes to the TJs (Benais-Pont et al., 2003). Its localization at the TJs is most likely regulated by its interaction with CGN and CGNL1 (Rouaud et al., 2020) (Figure 3). Importantly, depletion of either CGN or CGNL1 increases RhoA activity in both epithelial and endothelial cells (Aijaz et al., 2005; Guillemot et al., 2008; Tian et al., 2016; Holzner et al., 2021), suggesting that CGN and CGNL1 sequester GEF-H1 form RhoA within the TJ area or maintain GEF-H1 functionally inactive. In line with an inhibitory function of CGN and CGNL1 on RhoA activation, depletion of CGN in endothelial cells enhances the permebaility of endothelial cells induced by agonists such as thrombin or histamine concomitant with increased association of GEF-H1 with RhoA and increased RhoA-GTP levels, whereas ectopic expression of CGN protects endothelial cells from the effects of these agonists (Tian et al., 2016; Holzner et al., 2021). These observations are, thus, in line with a model that the binding of GEF-H1 to CGN or CGNL1 is required to inhibit RhoA activation at the TJs and prevent a loss of the barrier function. Interestingly, CGN and CGNL1 negatively regulate the expression levels of claudin-2 (Guillemot et al., 2013), and, similar to CGN and CGNL1, depletion of claudin-2 activates GEF-H1 and increases RhoA activity (Dan et al., 2019).

p114RhoGEF/ARHGEF18 is a GEF with high specificity for RhoA (Blomquist et al., 2000). It is also localized at the TJs and interacts with both cingulin (Terry et al., 2011) and PATJ (Nakajima and Tanoue, 2011) (Figure 3). Its depletion leads to a disorganized circumferential actomyosin belt as a result of reduced F-actin and myosin IIA accumulation along apical cell-cell junctions (Nakajima and Tanoue, 2011). Ectopic expression of CRB3 induces an epithelial phenotype in HeLa cells which is associated with recruitment of Pals1 and p114RhoGEF to cell-cell junctuions, formation of a cortical F-actin belt, and increased activities of RhoA and ROCK1/2 (Loie et al., 2015). These findings strongly suggest that p114RhoGEF activity is required to maintain the apical actomyosin organization. p114RhoGEF depletion also results in an impaired barrier function after Ca^2+^-switch-triggered junction formation (Terry et al., 2011). This observation indicates that p114RhoGEF is required during cell-cell contact and TJ formation (Terry et al., 2011). Of note, despite defects in lumen formation when cells are grown in a three-dimensional matrix after p114RhoGEF depletion, as indicated by multiple lumen formation, apico-basal membrane polarity is not grossly altered in these cells (Terry et al., 2011), suggesting that p114RhoGEF specifically regulates the gate function of TJs.

PDZ-RhoGEF/ARHGEF11 is a RhoA-specific GEF which has been found to localize at the TJs in polarized epithelial cells *in vitro* as well as *in vivo* (Itoh et al., 2012). PDZ-RhoGEF directly interacts with ZO-1 (Itoh et al., 2012) (Figure 3), and its localization at the TJs depends on ZO-1, strongly suggesting that ZO-1 serves as a scaffold for PDZ-RhoGEF at the TJs. PDZ-RhoGEF is required for the timely maturation of TJs and development of the barrier function after Ca^2+^ switch-triggered junction formation but its activity seems not to be required once mature TJs have been formed (Itoh et al., 2012). This suggests that PDZ-RhoGEF is primarily necessary during junction maturation and TJ formation. Its constitutive association with ZO-1 (Itoh et al., 2012) also suggests that it serves to regulate RhoA and myosin light chain (MLC) kinase activity in close spatial proximity of ZO-1, which is in agreement with the localization of ZO-1 at cell-cell junctions early on from the formation of pAJs to fully matured cell-cell junctions (Yonemura et al., 1995; Ando-Akatsuka et al., 1999). Its association with ZO-1 at the TJs, however, could also mean that PDZ-RhoGEF activity is necessary when TJ need to be repaired, for example after mechanical injury (see below). Studies in keratinocytes further indicated a role of an epithelial-specific splicing variant of PDZ-RhoGEF in the maintenance of TJs *via* RhoA activation and MLC phosphorylation (Lee et al., 2018).

### 3.6 Rho Small GTPases During Tight Junctions Remodeling

As opposed to the intuitive view of the TJs as a stable and rather unchanging barrier at the apical region of cell-cell contacts, individual molecular components of the TJs are remarkably dynamic (Shen et al., 2011). This is probably necessary to maintain the TJs in a regulatable condition and allows the tissue to adapt to changes in the environment, for example after physical damage, or in physiological situations that impose challenges to the maintenance of the barrier function and tissue integrity, such as cell division or cell extrusion. At the same time, however, an intrinsic dynamics bears the risk of interference by exogenous factors that might contribute to a loss of the barrier, cell-cell adhesion and eventually tissue integrity. Given that several regulators of Rho GTPase activity are localized at the TJs at steady state, it is conceivable that Rho GTPases are targeted during processes requiring TJ remodeling. RhoA seems to be particularly important for the maintenance of TJ integrity.

#### 3.6.1 RhoGTPases and Epithelial-Mesenchymal Transition

**FIGURE 4:**
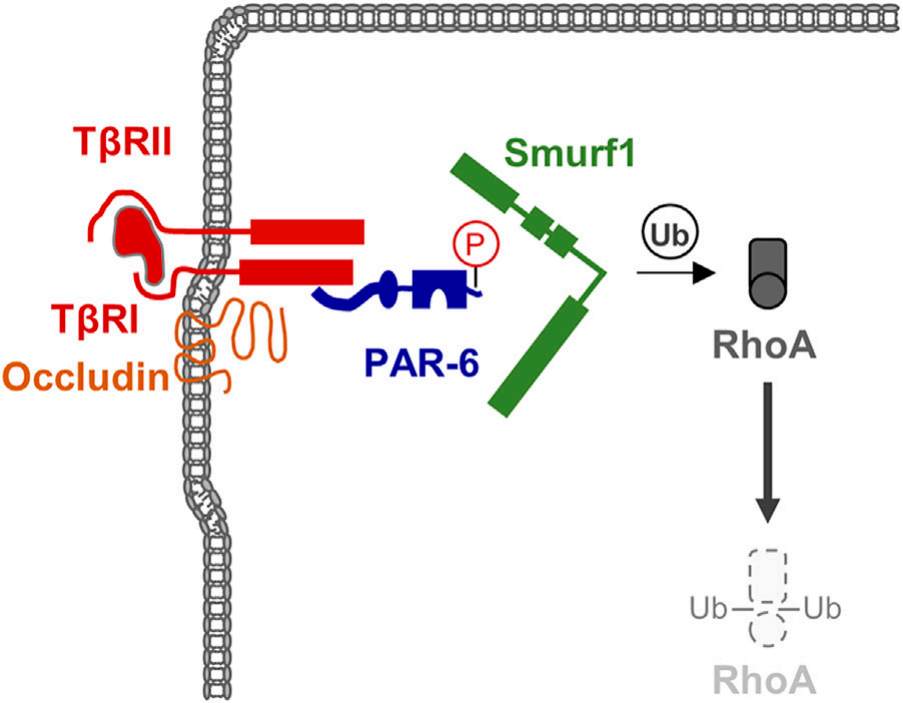
TGFβ signaling triggers RhoA degradation during EMT. TGFβ receptors are localized at theTJ through the interaction of TβRI with Occludin. TGFβ signaling triggers phosphorylation of PAR-6 resulting in the recruitment of the ubiquitin ligase Smurf1 and subsequent ubiquitination and degradation of the local pool of RhoA.

Epithelial-mesenchymal transition (EMT) is a cellular programme that allows epithelial cells embedded in an epithelial tissue to transdifferentiate into motile mesenchymal cells (Yang and Weinberg, 2008; Lamouille et al., 2014). A key event during EMT is the suppression of E-cadherin by a number of transcription factors, which supports the disassembly of cell-cell contacts and a loss of apico-basal polarity (Lamouille et al., 2014). Among the various signaling pathways identified to operate during EMT, TGFβ signaling has turned out as a key signaling pathway during EMT (Yang and Weinberg, 2008). TGFβ signaling targets RhoA at the TJs. By inducing TGFβ receptor I (TβR) I and TβR II dimerization at the TJs, TGFβ triggers PAR-6 phosphorylation, followed by recruitment of the ubiquitin ligase Smurf1, which ubiquitinates RhoA and thus targets RhoA for proteasomal degradation (Barrios-Rodiles et al., 2005; Ozdamar et al., 2005) (Figure 4). The TJ-specific targeting of RhoA activity by TGFβ further underlines the necessity of active RhoA at TJs to maintain TJ integrity. Importantly, TGFβ-activated transcription factors such as Snail repress the expression of a number of TJ-localized integral membrane and peripheral membrane proteins, including claudins, occludin and CRB3, and Pals1, PATJ, and PAR-3 (Lamouille et al., 2014), many of which are involved in Rho GTPase regulation (see above).

#### 3.6.2 RhoGTPases and Tight Junctions Repair—Rho Flares

**FIGURE 5:**
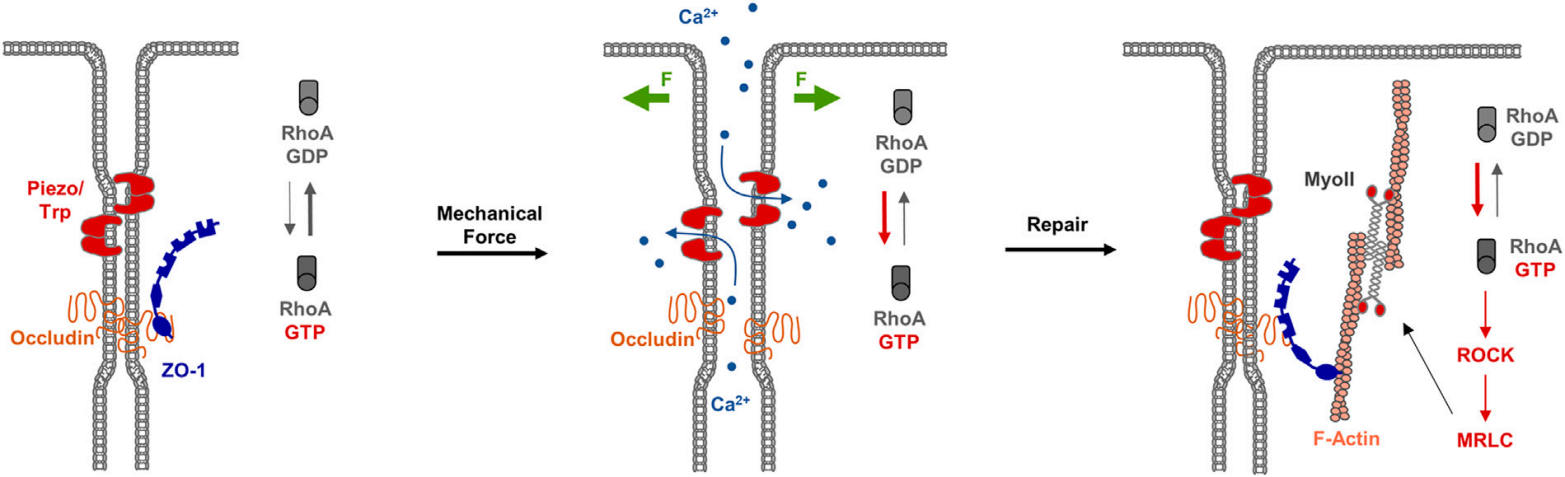
RhoA flares repair TJ breaches. Left panel: Under steady state conditions, TJs form a functional barrier that prevents the free diffusion of small solutes along the paracellular pathway. Mechanosensitive receptors like Piezo or Transient Receptor Potential (TRP) channels are inactive. Middle panel: Mechanical forces (F, green arrows) trigger the activation of Piezo / TRP mechanosensitive Ca^2+^ channels, which results in an influx of Ca^2+^ ions and the activation of the local pool of RhoA. Right panel: The local pool of active RhoA stimulates ROCK and MRLC resulting in actomyosi-based contractility and TJ repair.

During development, epithelial tissues frequently face challenges to the barrier function, for example during morphogenetic changes as they occur during gastrulation (Wallingford et al., 2001), or during cellular events like cell division (Fink et al., 2011) or cell extrusion (Kocgozlu et al., 2016). As recent observations during *X.laevis* gastrulation indicate, breaches at the TJs sporadically occur, which are shortlived and are rapidly repaired (Stephenson et al., 2019). A detailed investigation of the underlying mechanisms indicated that at sites of local TJ breaches, visualized with a sensitive tracer detection system, GTP-loaded RhoA rapidly accumulates and triggers local actin polymerization and acto-myosin-based contraction. At sites of TJ breaches markers like ZO-1 disappear, and they reappear shortly after the recruitment of active RhoA (Stephenson et al., 2019). This repair process is preceded by a local increase in the intracellular Ca^2+^ concentration, suggesting that mechanosensitive Ca^2+^ channels act as sensors of TJ breaches, and that a local increase in intracellular Ca^2+^ activates RhoA at the TJs (Varadarajan et al., 2022) (Figure 5). These findings thus provide strong evidence that in reponse to local perturbations of TJ integrity, RhoA is recruited and/or activated locally to induce contractility of the actin cytoskeleton to support the repair of TJs and to re-establish the epithelial barrier function.

## 4 Rab Family Small GTPases in Membrane Polarity

### 4.1 Rab Family GTPases in Polarized Vesicle Transport

Rab GTPases are central regulators of intracellular membrane trafficking involved in the biogenesis, transport and fusion of organelles and vesicles (for reviews see (Zerial and McBride, 2001; Novick, 2016; Pfeffer, 2017). Typically, they bind to specific organelle/vesicle membranes in their active, GTP-loaded conformation with specificity mediated by membrane-resident GEFs but also the Rab protein itself. The membrane-associated Rabs then serve as a platform for a large group of effector proteins that transmit functional specificity, e.g., by initiating vesicle budding at a donor organelle, mediating transport through direct or indirect interactions with microtubule or actin tracks, establishing tethers between membrane surfaces in the course of fusion events and links to the actual fusion machinery (for reviews see (Langemeyer et al., 2018; Lamber et al., 2019). Interestingly, Rab proteins can also provide directionality to membrane transport pathways by recruiting specific GEFs or GAPs for a downstream activation or an upstream inactivation of another family member. This Rab cascade has been well established for endosomal membrane trafficking where the progression from early to late endosomes is catalyzed by a conversion from Rab5 to Rab7, which itself is mediated by the recruitment of a Rab7 GEF through endosome-bound Rab5 (Rink et al., 2005; Poteryaev et al., 2010). Rab-mediated directional movement of vesicles that transport cargo from and to the plasma membrane is of particular relevance in polarized epithelial and endothelial cells where selective exocytotic transport and unique recycling pathways help establish and maintain the apical and basolateral plasma membrane compartments with their unique protein and lipid compositions.

### 4.2 Rab11 as a Central Regulator of Apical Delivery Pathways

Once a polarized state of an epithelium or endothelium characterized by the two principal membrane domains is established, it has to be maintained, among other things by directed transport of vesicular carriers from the post-trans-Golgi network (TGN) to these domains. Sorting motifs that define the transport of such carriers to either the apical or basolateral domain had been identified already early on. They include tyrosine-based motifs, e.g. found in the LDL receptor, directing a protein to the basolateral membrane, and glycosylations and GPI anchors routing the protein to the apical surface (for review see (Nelson and Yeaman, 2001). The cellular machinery mediating this selective transport involves coat proteins as well as membrane segregation and the formation of raft-like microdomains but is yet far from being fully understood.

Several Rab proteins function in the post-TGN transport to the different membrane domains in polarized epithelial and endothelial cells. A central role has been described for Rab11, which has been linked to the transport of apically destined proteins and vesicles in polarized cells. Examples are the apical delivery of the sodium/hydrogen exchanger 3 (NHE3) and the cystic fibrosis conductance regulator (CFTR) in human intestinal epithelial cells, and the apical exocytosis of discoidal/fusiform-shaped vesicles (DFVs) in bladder umbrella cells (Khandelwal et al., 2008; Vogel et al., 2015). These observations analyzing apical trafficking in fully polarized cells are in line with the role of Rab11 in the *de novo* specification of an apical membrane domain in the course of tubular morphogenesis. Often using MDCK cyst formation as a model for polarization in 3D, several studies have identified a crucial role for Rab11 (Bryant et al., 2010); for reviews see (Apodaca et al., 2012; Jewett and Prekeris, 2018). Here, early specification of an apical membrane domain already begins during cell division, when endosomal vesicles are recycled in a polarized manner. During cytokinesis, the vesicles are directed to the cleavage furrow which forms at the site of the midbody, a microtubule-rich structure that marks the location of future lumen formation and that is positive for several Rab proteins including Rab8, Rab11 and Rab35 (Bryant et al., 2010; Klinkert et al., 2016).

**FIGURE 6:**
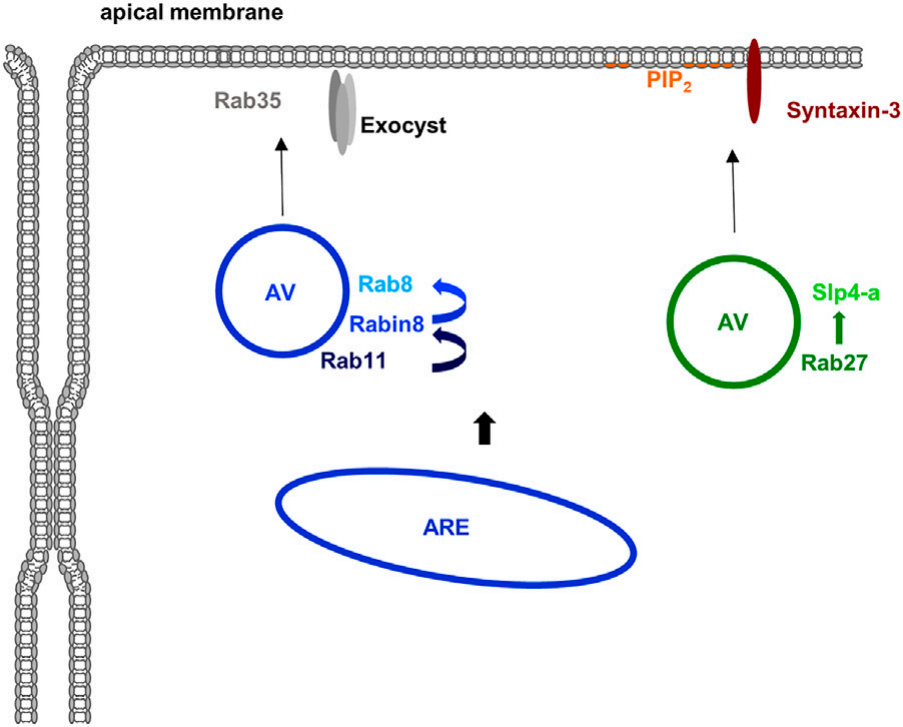
Rab proteins in apical membrane traffic. Several Rab proteins are known to participate in the delivery of material to the apical membrane domain in polarized epithelial cells. Apically destined vesicles (AV) contain Rab11 which recruits the Rab8 GEF Rabin8 thereby activating Rab8. Rab35 and the exocyst complex are most likely involved in the apical membrane targeting of these vesicles. Another class of apically destined vesicles operating in the formation of an apical lumen in 3D are positive for Rab27 (and most likely also Rab3 isoforms). The Rab27 effector Slp4-a is targeted to these vesicles and most likely functions in conjunction with Slp2-a to tether the vesicles at PtdIns(4,5)P_2_-rich apical membrane domains with fusion eventually mediated by syntaxin-3. Most likely, both types of vesicles originate at the apical recycling endosome (ARE) although formation at the TGN is also feasible at least for the Rab27 positive vesicles.

Rab11, a well known Rab of recycling endosomes, triggers a Rab cascade by recruiting a GEF for Rab8 (Rabin8) which in turn activates Rab8 (Figure 6). The Rabs, both known to interact with the plasma membrane-associated and fusion-promoting exocyst complex (Zhang et al., 2004; Wu et al., 2005), together with other components including the Cdc42 GEF Tuba then assemble at an apical membrane initiation site (AMIS) where lumen formation occurs (Bryant et al., 2010). In the order of these events, the Rab11/8 vesicles transition an apical (recycling) endosome which is also found in fully polarized cells where it organizes endosomal recycling pathways to the (established) apical membrane (see below). The exocyst complex likely has multiple additional functions in the establishment and maintenance of epithelial polarity. For instance, it is involved in the clustering of E-cadherin and the proper formation of adherens (Yeaman et al., 2004; Xiong et al., 2012) and also localizes to tight junctions where it participates in TJ formation through interactions with RalGTPases (Hazelett et al., 2011), which also regulate basolateral protein traffic (Moskalenko et al., 2002), and is interacting with PAR-3 (Ahmed and Macara, 2017). Due to the focus of this review on Rho and Rab GTPases these exocyst functions are not discussed in detail and the interested reader is referred to other reviews on this topic (Wu and Guo, 2015; Polgar and Fogelgren, 2018).

### 4.3 Other Rab Proteins in Apical Exocytosis

In addition to Rab8 and Rab11, other Rab proteins have been described to participate in the delivery of material to the apical membrane of polarized epithelial and endothelial cells. They include Rab3 and Rab27 isoforms which have been implicated in the early specification of the apical membrane domain during lumen formation. Both have been identified on vesicles that deliver material to the apical membrane to initiate lumen formation. Their effectors, synaptotagmin-like protein (Slp) 2-a and Slp4-a are mediating this event by linking the vesicles to the PtdIns(4,5)P_2_ -rich apical membrane and promoting tethering and fusion in conjunction with the t-SNARE syntaxin-3 (Galvez-Santisteban et al., 2012) (Figure 6). Further supporting a role of this Rab27/Slp2/4-centered network in establishing/maintaining epithelial polarity, mutations in syntaxin-3 have been identified in patients suffering from microvillus inclusion disease, a congenital enteropathy that is characterized by disturbed polarity of intestinal epithelial cells which show a loss of brush borders and a subapical accumulation of vesicles (Cutz et al., 1989). Interestingly, these vesicles are positive for Rab11 and Rab8 indicative of a perturbed apical recycling compartment. Together with the observation that the exocyst-binding Rab8 is also present on the apical Rab27 vesicles these findings suggest a connection between the Rab11 and Rab27 centered pathways of lumen initiation (for review see (Jewett and Prekeris, 2018; Polgar and Fogelgren, 2018). Yet another Rab implicated in the specification of the apical membrane and the initiation of lumen formation is Rab35. It localizes to apical vesicles and Rab35 depletion leads to multiple lumen formation in the MDCK cyst assay (Klinkert et al., 2016; Mrozowska and Fukuda, 2016).

Several components described to be involved in apical membrane transport and lumen formation in epithelial cells, in particular Rab3 isoforms, Rab27a and Rab35, have also been identified as regulators of the exocytotic delivery of specialized secretory granules in endothelial cells. In their mature form these lysosome-related organelles, the so-called Weibel-Palade bodies, are preferentially secreted at the apical membrane of the polarized endothelium in a process that is induced by endothelial cell activation and that involves Rab3b/d and Rab27a and their effectors MyRIP and Slp4-a (for review see (McCormack et al., 2017; Schillemans et al., 2019; Nass et al., 2021). A related Rab27 isoform, Rab27b, was identified on subapical vesicles in lacrimal gland acinar cells, and by expression of dominant active and inactive mutant proteins as well as knock-out mouse studies was reported to participate, possibly in conjunction with Rab3D, in the formation and apical release of secretory vesicles in these cells (Chiang et al., 2011). DFVs of umbrella bladder cells (see above) are another pool of subapical vesicles positive for Rab27b. The apical exocytosis of these vesicles is triggered by filling of the bladder and was shown to be inhibited by depletion of Rab27b, but not Rab27a. Interestingly, the Rab27b-dependent regulation of DFV exocytosis appears to operate in parallel to the Rab11-Rab8 pathway discussed above, as Rab27b depletion has no effect of the Rab11-positive parameters (Gallo et al., 2018). Although less well characterized, Rab17 also appears to regulate the apical delivery of exocytotic vesicles, as shown for transcytosis in polarized hepatic WIF-B cells (Striz et al., 2018).

### 4.4 Rabs in Basolateral Transport

As compared to the apical delivery of transport vesicles in polarized epithelial and endothelial cells, much less is know about the involvement of Rab proteins in basolateral trafficking, also because this had long been considered the default route of post-TGN traffic. Indirect evidence based on localization suggests that Rab13 could be involved in basolateral membrane transport in polarized osteoclast (Hirvonen et al., 2012) and in polarized Drosophila follicle cells, Rab10 is required for the secretion of basement membrane at the basal surface (Lerner et al., 2013). Rab10 was also identified in polarized MDCK cells to support the biosynthetic transport of basolateral cargo (Schuck et al., 2007) and to affect basolateral endocytic sorting/recycling pathways (Babbey et al., 2006). Moreover, in the *C. elegans* intestinal epithelium Rab10 was shown to participate in the formation of an endosomal tubular network required for the efficient recycling of cargo that is subject to clathrin independent internalization (Chen et al., 2014). Together these observations suggest that Rab10 could regulate transport routes between basolateral sorting and recycling endosomes and thereby also exocytotic delivery of certain cargo to the basolateral membrane domain.

### 4.5 Rabs in Endosomal Recycling in Polarized Cells

The above considerations indicate that biosynthetic post-TGN transport routes and endosomal recycling pathways are tightly interlinked also in polarized cells. Endocytic recycling is required both during establishment of the two distinct membrane domains of polarized cells but also when they have to be maintained in fully polarized tissues. For example, newly synthesized material (proteins, lipids) that is delivered to the basolateral domain either by default or mistake but is destined for and functions at the apical domain has to be re-internalized and then delivered to the correct membrane domain. A paradigm for the analysis of these trafficking routes has been the glycoprotein podocalyxin (PCX) initially identified in renal podocytes but present in the apical glycocalix of many epithelia and endothelia. The transcytotic endosomal recycling of podocalyxin involves an internalization from the basolateral membrane, transport through basolateral early/sorting endosomes and delivery to the Rab11 positive apical recycling compartment (for review see (Roman-Fernandez and Bryant, 2016). A comprehensive analysis of Rab GTPases involved in podocalyxin trafficking in epithelial cells was performed by Mrozowska and Fukuda (Mrozowska and Fukuda, 2016) who studied the transport in MDCK cells cultivated to polarize in 2D (epithelial sheet formation) and 3D (luminogenesis and cyst formation). Using a combination of colocalization and knockdown screenings they could show that the majority of Rabs are involved and function at different stages of the PCX transcytosis in both 2D and 3D conditions but that some of them appeared to be primarily engaged in either the 2D (Rab13 and Rab14) or 3D cultures (Rab4, Rab15, Rab19, Rab25). An interesting finding concerned Rab35 which participated in the polarized PCX transport both in 2D and 3D but engaged different effectors, the inositol polyphosphate 5-phosphatase OCRL in 2D monolayers, and the Arf GAP ACAP2 in 3D cysts. This supports the notion that transcytosis and endosomal recycling of apical membrane proteins in polaized epithelial cells is rather complex, is context dependent (2D vs 3D). and is intricately regulated by different Rab protein family members.

## 5 Conclusion

Epithelial cells and endothelial cells develop a highly pronounced apico-basal polarity to form membrane compartments with distinct functions. Rho family GTPases contribute to the development of membrane polarity by regulating the formation as well as the maintenance of TJs. Rab family GTPases contribute to membrane polarity by regulating the trafficking and delivery of vesicles and cargo to distinct membrane compartments. The involvement of Rho and Rab small GTPases and in particular the increasing number of Rho GEFs and GAPs at the TJs indicates that the generation of membrane polarity is a sophisticated and dynamically regulated process. Evidence accumulates suggesting that the gate function and the fence function of TJs are regulated through distinct Rho GTPase-based mechanisms. Given that the molecular mechanism underlying the fence function is still largely unknown, it will be important to understand in more detail the site-specific regulation of Rho small GTPases through their interaction with GEFs, GAPs and GDIs at the TJs.
